# Susceptibility of Enamel Treated with Bleaching Agents to Mineral Loss after Cariogenic Challenge

**DOI:** 10.1155/2011/953835

**Published:** 2011-08-01

**Authors:** Hüseyin Tezel, Cigdem Atalayin, Ozlem Erturk, Ercument Karasulu

**Affiliations:** ^1^Department of Restorative Dentistry and Endodontics, Faculty of Dentistry, Ege University, Bornova, 35100 İzmir, Turkey; ^2^Department of Biopharmaceutics and Pharmacokinetics, Faculty of Pharmacy, Ege University, Bornova, 35100 İzmir, Turkey

## Abstract

*Objectives*. Controversial reports exist whether bleaching agents cause a susceptibility to demineralization. The aim of this study was to compare the calcium loss of enamel treated with different bleaching agents and activation methods. *Method and Materials*. The specimens obtained from human premolars were treated in accordance with manufacturer protocols; 10% carbamide peroxide, 38% hydrogen peroxide light-activated, 38% hydrogen peroxide laser-activated, and no treatment (control). After cariogenic challenge calcium concentrations were determined by Inductively Coupled Plasma Mass Spectrometry. *Results*. No differences were found between the calcium loss of the laser-activated group and 10% carbamide peroxide group (*p* > 0.05). However, the differences between laser-activated and control groups were statistically significant (*p* < 0.05). The differences between 10% carbamide peroxide and the control group were not significant (*p* > 0.05). On the other hand, the light-activated group showed a significantly higher calcium loss compared with the other groups (*p* < 0.05). *Conclusions*. The results show that bleaching agents may cause calcium loss but it seems to be a negligible quantity for clinical aspects.

## 1. Introduction

In the last century, bleaching of discolored teeth has attracted a lot of attention and continues to gain popularity in public. Patients and consumers continue to demand not only a healthy mouth, but also a perfect appearance. There has been a rapid development of nonrestorative treatment for discolored teeth. The application of different concentrations of hydrogen peroxide (HP) or carbamide peroxide (CP) to the enamel surface results in a whiter tooth color shade. This procedure can be considered as a conservative approach toward obtaining esthetic or cosmetic results compared with other methods such as veneering or crowning.

Bleaching of vital teeth can be achieved by the use of a custom made; vacuum-formed appliance with lower concentrated bleaching gels (10% HP or 10–20% CP) termed “home bleaching.” Another method is applying higher concentrations of bleaching gels (30–38% HP or 35–37% CP) directly onto the tooth surface in the dental chair, which is termed “in-office bleaching” [1, 2]. It is known that 10% CP has an equivalent strength to 3.6% HP and that it has been approved by the FDA as the safe concentration for bleaching [3]. This technique can be also considered as a milder method compared to the in-office techniques. 

The decomposition of hydrogen peroxide results in oxygen and perhydroxyl free radicals, which then oxidize the stained macromolecules and break them down into smaller fragments. Then, the fragments diffuse across the tooth surface, resulting in the bleaching effect [1, 4]. To accelerate this reaction, heat, lights, and lasers have been used but today lights and lasers are the preferred activation methods. The use of activation methods has shortened the extensive period of time, which involves the direct contact of the high concentrated bleaching agents with the tooth surface that may cause a certain amount of enamel matrix degradation. A shortened treatment period may eradicate the side effects of high concentrated HP [5, 6]. 

Studies have shown that bleaching agents can cause structural alterations on the enamel surface and that the biomechanical properties of the enamel can change [7–17]. Additionally, *in vitro* studies have shown a close correlation between the bleaching agent effects and the enamel surface changes [18]. There are also some reports that bleaching agents promote chemical and microstructural changes in enamel, similar to initial caries lesions [16, 19], but it has been noted that these alterations have no clinical significance. Basting et al. [20] noted that bleaching agents may have a possible influence on active caries lesions in enamel and dentin. It is a question whether the enamel would be more susceptible to cariogenic challenge after the bleaching process. Little is known about this issue. The controversial results of existing reports and the continuous appearance of new bleaching products and light activation units that are on the market demand more research in this field.

Demineralization is a process which involves the loss of calcium ions of the surface of calcified dental tissues. In a favorable oral environment, the loss of calcium (demineralization) is balanced with the uptake of calcium (remineralization) from the tooth's microenvironment. Calcium losses of calcified structures are commonly measured by an atomic absorption spectrophotometer. Another method, the Inductively Coupled Plasma-Mass Spectrometer (ICP-MS), is a type of mass spectrometry that is highly sensitive and capable of the determination of a range of metals and several nonmetals at concentrations below one part in 10^12^. It is based on coupling together an inductively coupled plasma as a method of producing ions (ionization) with a mass spectrometer as a method of separating and detecting the ions. ICP-MS is also capable of monitoring isotopic speciation for the ions of choice [21, 22].

The purpose of this *in vitro* study was to compare the Ca^2+^ loss of enamel treated with 10% CP, 38%  HP activated with light, and 38% HP activated with diode laser which had different contact times according to the manufacturers' instructions using an Inductively Coupled Plasma-Mass Spectrometer.

## 2. Method and Materials

**Figure 1:**
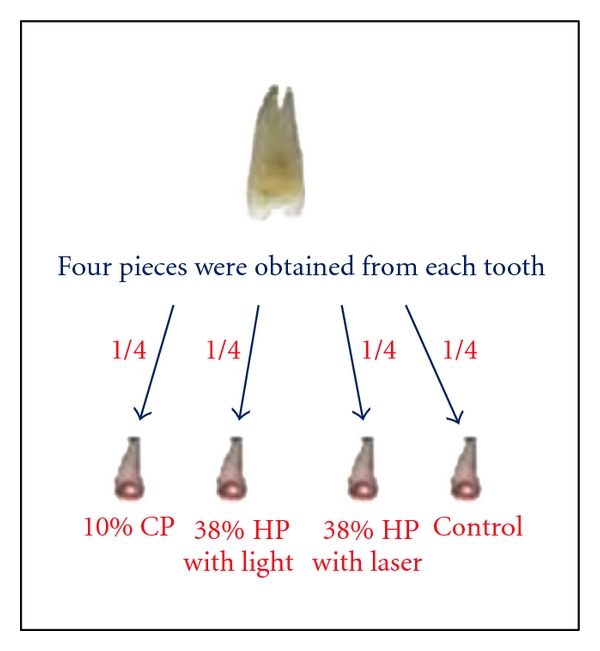
The tooth was divided buccolingually or buccopalatinally into two parts and these halves were then sectioned longitudinally into two parts. Four specimens were obtained from each tooth.

Human premolars extracted for orthodontic purposes were rinsed in tap water and were cleaned of plaque and debris with a dental handpiece and brush. The buccal, lingual, and occlusal surfaces were checked under a stereomicroscope, and teeth with enamel defects or cracks were rejected. The selected ten teeth were stored in 0.9% saline solution for one week and then rinsed in distilled water. Each tooth was sectioned buccolingually or buccopalatinally into two halves with a diamond disc. These halves were then sectioned longitudinally into two parts, so that four specimens were obtained from each tooth. These specimens were randomly assigned to one of the four groups, ensuring that each part of every specimen would be in a different group, which is one of the four groups. Teeth were then covered with wax except for the enamel surface (Figure 1). The size of the window on the exposed enamel surface was about 4 × 6 mm.

Three of the groups were then treated with bleaching agents; 10% CP (Opalescence PF 10% CP, Ultradent Products Inc., South Jordan, USA) (Group 1, *n* = 10/group), 38% HP (WHITEsmile XTRA 38% HP, Germany) activated with halogen light (Optilux 501, Kerr, USA) (Group 2, *n* = 10/group), and 38% HP (WHITEsmile XTRA 38% HP, Germany) activated with 810 nm wavelength diode laser (Group 3, *n* = 10/group), respectively, according to manufacturers' instructions (Table 1). The specimens in the fourth group (Group 4, *n* = 10/group) were used as a control group; no agent was used and they were kept in artificial saliva [23–25] consisting of 0.7 mmol/L CaCI_2_, 0.2 mmol/L MgCI_2_, 4 mmol/L KH_2_PO_4_, 30 mmol/L KCI, and 20 mmol/L serum hepes (pH = 7) during the test period. 

Group 1 was treated with 10% CP in a custom-made vacuum-formed tray (*n* = 10/group). The specimens were placed on the anterior side of the phantom jaw in such a way that the enamel surfaces would face the buccal side. Stone casts were obtained from the impression made from a silicone impression material with type IV dental stone (Die-Keen; Heraeus Kulzer Inc., South Bent, Ind.). The enamel parts on the buccal surfaces of the casts were blocked out by a resin creating reservoirs. A vacuum tray was formed out of the soft tray sheet which had a thickness of 0.5 mm. 10% CP (Opalescence PF 10% CP, Ultradent Products Inc., South Jordan, USA) was added into this bleaching tray and was kept there for 8 hours a day throughout 14 days. Following every session, the teeth were rinsed, dried, and topical fluoride agent (WHITEsmile after bleaching mousse, 30% xylitol, 4.2% potassium nitrate, 1450 ppm sodium fluoride, Germany) was applied for ten minutes. The specimens were kept in artificial saliva during the test period to take advantage of the remineralization action of saliva. The artificial saliva used in this study was freshened every day to be able to obtain constant ion concentration.

In Group 2, a bleaching agent consisting of 38% HP (WHITEsmile XTRA 38% HP, Germany) was used according to the manufacturers' instructions with light activation (*n* = 10/group). The gel was applied to enamel surfaces of the specimens at about 1–1.5 mm thickness and activated with halogen light on bleaching mode (Optilux 501, Kerr, USA) for 30 seconds. The gel was replaced three times. After removing the whitening gel, the teeth were rinsed and dried and the same topical fluoride agent (WHITEsmile after bleaching mousse, 30% xylitol, 4.2% potassium nitrate, 1450 ppm sodium fluoride, Germany) was applied for ten minutes. The specimens were kept in artificial saliva until the following day. This procedure was repeated every other day for 3 days of application. 

Group 3 was treated with 38% HP (WHITEsmile XTRA 38% HP, Germany) with 810 nm wavelength diode laser (*n* = 10/group). The bleaching gel was applied as a layer (~1–1.5 mm) to the enamel surfaces of the specimens. To have the optimum effectiveness, the bleaching gel was activated with 810 nm wavelength diode laser (LaserSmile, Biolase, USA) using the whitening program (10 Watt-continuous mode for 15 seconds, with 1 mm distance from the bleaching gel). After each activation, the gel was agitated and the activation was repeated with one-minute intervals. This was repeated for four times. The application was repeated three times for every session. At the end of the application, the teeth were rinsed and dried and the same topical fluoride agent (WHITEsmile after bleaching mousse, 30% xylitol, 4.2% potassium nitrate, 1450 ppm sodium fluoride, Germany) was applied for ten minutes. The specimens were kept in artificial saliva until the following day. This procedure was repeated 3 times every other day.

The specimens in the fourth group were used as controls and kept in artificial saliva during the test period.

Immediately after the application of the bleaching agents for the prescribed time, the specimens were rinsed with a water spray and dried with blasts of air. Then, the enamel was covered with wax so as to expose a round window area (6.83 mm^2^) and acetic acid buffered with 0.34M sodium acetate (pH = 4) was used as an artificial caries solution [17, 26, 27]. Salt of calcium monohydrate [Ca(H_2_PO_4_)_2_.H_2_O)] was dissolved to obtain 10 mmoL Ca^2+^ and 20 mmoL PO_4_^3−^ in the solution [17, 26, 27].

**Figure 2:**
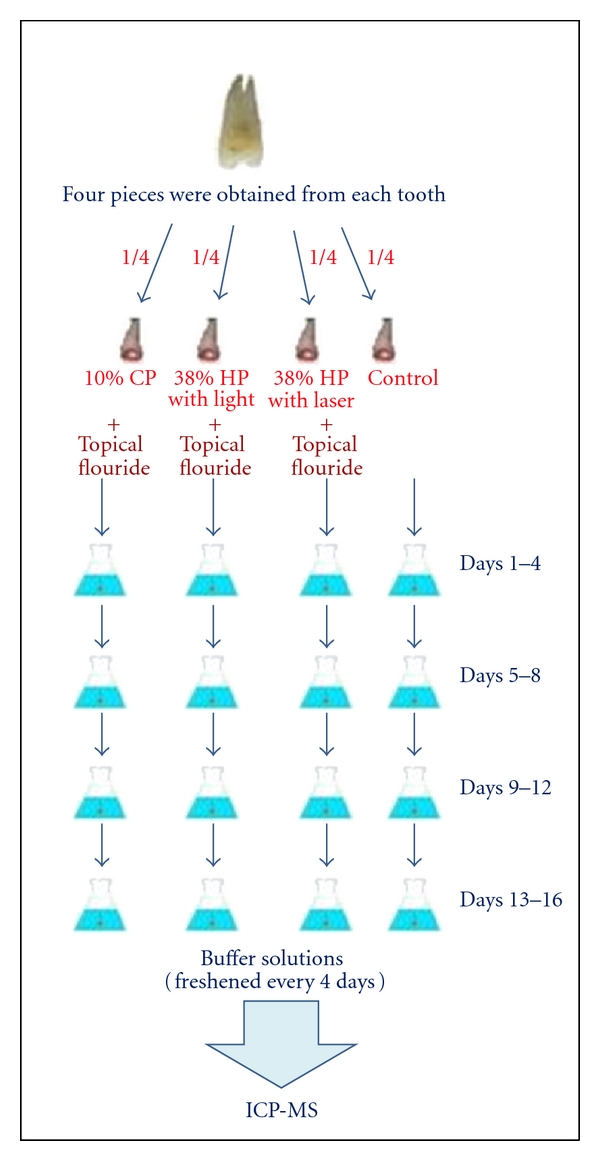
The specimens were treated with the buffer four times every four days, in 16 days. The solutions in the tubes were tested for their Ca^2+^ loss with Inductively Coupled Plasma-Mass Spectrometry (ICP-MS).

Each specimen was treated with 50 mL of solution in the polyethylene test tubes. The specimens were treated with the buffer four times every four days, in 16 days. Each day, the test tubes were agitated. In the end of the forth day, each specimen was taken out of the test tube and placed in new tubes, containing fresh buffer solution. The previous solutions were kept in their tubes to be tested afterwards for their Ca^2+^ loss with inductively coupled plasma mass spectrometry (ICP-MS) (Agilent 7500 ce, Inductively Coupled Plasma-Mass Spectrometer, Octopole Reaction System) (Figure 2).

Repeated measures ANOVA was performed for calcium loss with both factors days (4th, 8th, 12th, and 16th) and groups (10% CP, 38% HP with light activation, 38% HP with laser activation, and control). The analysis was performed with Post-hoc tests including Bonferroni and Dunnett *C* test.

## 3. Results

**Figure 3:**
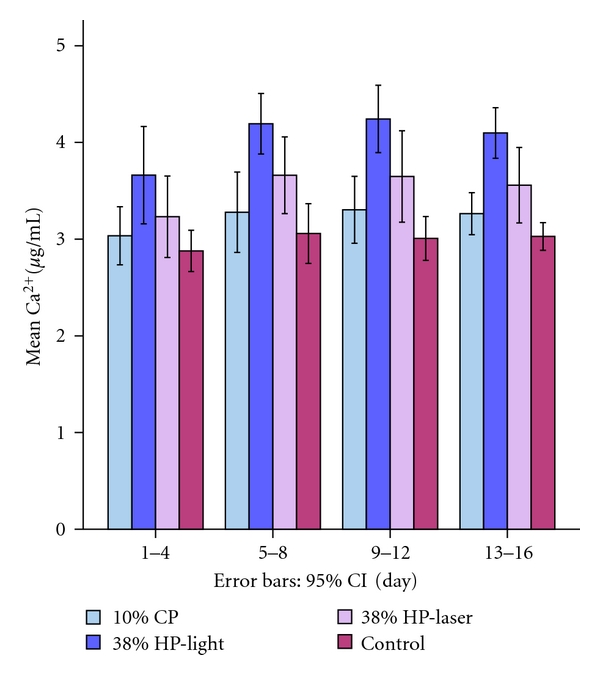
Ca^2+^ values measured in the buffer solution after application of bleaching agents.

The calcium concentrations of the samples measured at end of the 4th, 8th, 12th, and 16th days are shown in Figure 3. 

The loss of calcium in each of the test groups was compared with that of the control group using the repeated measures ANOVA. A statistically significant difference was observed among the groups on days 4, 8, 12, and 16 and in total (*p* < 0.05). 

Because the ANOVA test was found to be significant, post-hoc test was used to analyze the significance between the groups. Dunnett *C* test was used to analyze the heterogeneous distribution between 9–12th days whereas Bonferroni test was used for the other remaining homogeneous groups (Table 2). 

**Figure 4:**
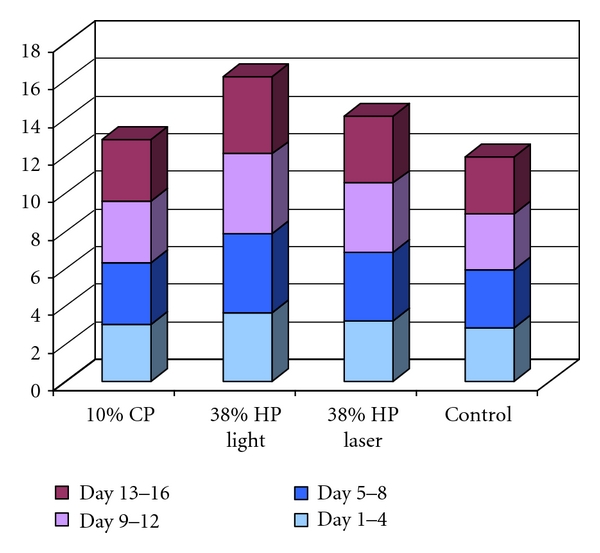
Release of Ca^2+^ after treatment with bleaching agents (per mm^2^, measured cumulatively).

At the end of the 16th day, Calcium ions released per mm^2^ (Figure 4) were calculated cumulatively as follows:

10% CP group: 12.88 ± 1.48 *μ*g/mL,
38% HP with light activation group: 16.20 ± 1.67 *μ*g/mL,
38% HP with laser activation group: 14.10 ± 2.16 *μ*g/mL,
control group: 11.97 ± 0.87 *μ*g/mL.

No differences were found between the calcium loss of the laser-activated group and 10% CP group (*p* > 0.05). However, the differences between laser-activated and control groups were statistically significant (*p* < 0.05). The differences between 10% CP and the control group were not significant (*p* > 0.05). On the other hand, the light-activated group showed a significantly higher Ca^2+^ loss compared with the other groups (*p* < 0.05).

## 4. Discussion

Discoloration of permanent anterior teeth is an esthetic problem. Although there are different aesthetic values of the teeth, the shade of the teeth is the easiest to alter to gain cosmetic improvement. While an attractive smile has become an important component of the oral health, the demand of white teeth has brought the manufacturers to improve the nonrestorative treatment of discolored teeth. Today bleaching is an easy, conservative method to improve the esthetic appearance compared with other methods such as veneering or crowning [28, 29]. A follow-up of 30 patients who applied 10% CP revealed that 43% perceived their tooth color as stable 10 years after bleaching [30]. Swift et al. [31] examined the effects of the 10% CP which was used nightly for 2 weeks and found that the teeth were eight shade units lighter on the Vita shade guide on an average. While considering the fact that bleaching agents may alter the enamel, the conservative improvement with the bleaching process should not be undervalued.

Bleaching of vital teeth can be mainly divided into two concepts; the usage of lower concentrations of CP on custom-made trays at home and the usage of higher concentrations of HP in the dental office. The decomposition of these peroxides results in free radicals. These free radicals break down large pigmented molecules in enamel into smaller, less pigmented molecules through either oxidation or reduction reactions [1, 4].

While some studies [7–17] suggest that these oxidation reactions cause alterations on the enamel structure, other studies [32, 33] assume that, bleaching agents have no adverse effects on the tooth structure. Still, this issue is a debate.

Studies have shown that different products consisting of 10% CP do not adversely affect the enamel surface [32]. McCracken and Haywood [34] showed that teeth which were exposed to 10% CP lost calcium but mentioned that this amount was small and may not be clinically significant. Goo et al. [35] reported that the mineral content and the Ca/P ratio decreased after bleaching with 10% CP, but they concluded that these changes were negligibly different from the control groups. The findings of this study confirm the results of our previous study [17] and all the before mentioned studies. 

The physical and chemical soundness of the enamel depends on the pH and the saliva consisting of calcium phosphate and flouride. Caries lesions develop with the fermentation of carbohydrates by bacteria's, the formation of organic acids, and the pH decrease. The critical pH value for the enamel is pH = 5.5, and when the oral pH decreases below this value, the bands between the fibrils and apatite of the enamel dissolve and the inorganic structure is affected [36].

In a study [37], the enamel surface of bleached teeth was examined with a scanning electron microscope and a profilometer. The results showed that the enamel surface was affected by different concentrated bleaching agents but these differences were not related with the pH values of the agents. The pH values of the bleaching agents used in our study were measured with a pH meter and determined approximately pH = ~8 for each group. The pH values of bleaching materials were almost similar in this study, but the calcium losses of the groups were found to be different. For this reason, we think that the pH values of the bleaching agents may not be effective for the calcium loss of the groups and that these results are compatible with the study mentioned above.

Studies [7–18] report different results about the alterations caused by the bleaching agents; it is still advised to take protective precautions to avoid adverse effects. The saliva is capable of remineralizing and giving protective benefits during the bleaching process. During the study, the specimens were immersed in artificial saliva to reflect the oral conditions. Also, an after bleaching mousse containing flouride and potassium nitrate was applied for ten minutes to maintain the clinical schedule. This procedure gave us the potential effects of the topical flouride agent for remineralization. Fluoride has been admitted to remineralize softened enamel by increasing resistance to acid attacks by forming a calcium fluoride layer to inhibit demineralization [38–40]. It accumulates in the plaque fluid and as calcium fluoride on the enamel surface. During the acid challenge, calcium fluoride is dissolved [41]. It may be a question if the calcium loss could be from the dissolved calcium fluoride. Fundamentally, the source of calcium for calcium fluoride is from the enamel. Depending on this fact, the measured calcium loss after the acidic challenge should be from the enamel either directly or indirectly from the dissolved calcium flouride. Nevertheless, further studies are required to estimate this fact. Although all precautions were taken, demineralization differences were observed between the control group and the other groups. It is known that, as topical fluoride is applied following bleaching, the mineral loss is significantly reduced [42]. For this reason, it is still necessary to minimize the risk of even minor damage caused by these agents; it should be advised to take different precautions. 

Hegedüs et al. [43] showed that high concentrated HP had a more pronounced effect on enamel than did CP and a low concentrated HP. Bistey et al. [44] recommended the short time usage of bleaching agents and reported that lower peroxide concentrations could be safer than higher concentrated ones. They also noted that above a “critical” concentration, the destruction in enamel is not increased and concluded that the changes in enamel were directly proportional to the treatment time and peroxide concentration. Laser-activated bleaching offers an improvement in terms of effectiveness, short impact time, and enamel surface protection [5, 6]. The results of this study showed that the 38% HP activated with either light or laser caused more Ca^2+^ loss compared with the control and 10% CP group. But only the laser-activated 38% HP group had no statistically significant difference from the 10% CP group. During the study, the bleaching agents were used in accordance with manufacturers' protocols and these were followed also for the activation methods used. The aim was to follow the clinical protocol. According to the manufacturers' instructions, the chemical bleaching without activation is 15 minutes for three times. In total, the contact time for the bleaching gel throughout the study would have been 135 minutes. Activating the bleaching gel with halogen light reduced the contact time to 45 minutes and for the laser activated group to 36 minutes. It can be assumed that the higher concentration of HP could have caused more Ca^2+^ loss than the CP group, but due to laser activation which shortened contact time of the high concentrated bleaching gel, the calcium loss in the laser group was close to the CP group. 

Tezel et al. [17] researched the effects of different bleaching agents with different concentrations on Ca^2+^ loss from enamel. It has been concluded that higher concentrations of HP resolve more Ca^2+^ from the enamel surface. Thus, more changes may occur on enamel structure as the concentrations of bleaching agent increase.

The use of high-intensity light has been indicated for acceleration of the rate of chemical bleaching; however, it is not known whether light irradiation can promote additional effects on enamel surfaces. Zhang et al. [45] suggested that KTP laser is effective at providing brighter teeth and that it induced a safer pulpal temperature. Another study [46] noted a greater penetration of hydrogen peroxide into the pulp chamber as a result of activation by laser or LED. de Magalhães et al. [47] observed no change in enamel microhardness after treatment with hydrogen peroxide gel photoactivated using diode laser with or without fluoride application but that there was an increase in microhardness when enamel was treated only with fluoride photoactivated using a laser. Controversially it was reported that the bleaching treatments in combination with light irradiation or not can reduce the mineral content of enamel surface. It was noted that light irradiation increased the calcium loss for Whiteness HP Maxx but that there were no effects observed for Pola Office and Opalescence Xtra [48]. 

There is a linear relationship between the decrease in enamel microhardness and Ca^2+^ loss [49]. 3.95 *μ*g of calcium loss in a surface per mm^2^ means that the surface cannot be remineralized [50].

Although we did not research the effect of the bleaching agents on enamel microhardness, many studies [7, 51–54] reported that 10% CP caused no changes in the microhardness of the enamel whereas another study [55] demonstrated reduction in the microhardness of the enamel and dentin when only HP was used. Further research is advised to clarify this issue.

In this study, an up-to-date method called Inductively Coupled Plasma-Mass Spectrometry (ICP-MS) was used to measure the calcium loss of the bleached enamel specimens. Although atomic absorption spectrophotometer is a commonly used method to measure the Ca^2+^ loss of calcified tissues, we preferred to use ICP-MS, because it is highly sensitive and capable of determining very low concentrations of ions. This method is usually used for the analysis of biological materials including teeth [21, 22].

## 5. Conclusion

Considering the conditions tested, the changes in enamel were directly proportional to the treatment time and peroxide concentration. According to the methodology used in this study, higher concentrations of HP cause more Ca^2+^ loss than lower concentrations. The contact time of high concentrated bleaching agents may also be an important factor for Ca^2+^ loss. A recommendation to use activation methods which shorten the contact time of the highly concentrated bleaching agents can be used in the dental office. 

The findings of this *in vitro* study may not be full representative of the *in vivo* condition; in which the oral cavity is continually bathed with saliva that contains various minerals (*i.e.*, fluoride, calcium phosphate), lipids, carbonhydrates and proteins. They also do not represent unfavorable conditions where the deficiency of saliva or poor oral hygiene that might increase the caries risk. Further studies are needed to clarify the effects of these materials on Ca^2+^ loss of enamel and caries susceptibility. The highly sensitive Inductively Coupled Plasma-Mass Spectrometry is capable to determine low concentrations of ions. It is questionable if this amount of calcium loss in the bleached enamel would have a clinical relevance. In the limitations of this study, the results show that bleaching agents may cause calcium loss but it seems to be a negligible quantity for clinical aspects.

## Figures and Tables

**Table 1:** Test groups (*n* = 10 each group).

Bleaching agent	Product name	pH	Application	Application time
Group 1: 10% CP	Opalescence PF 10% CP, Ultradent Products Inc, South Jordan, USA	~8	Home	8 hours a day, 14 days
Group 2: 38% HP with light activation	WHITEsmile XTRA 38% HP, Germany	~8	In-office (with light activation)	15 minutes every session, total 3 sessions
Group 3: 38% HP with laser activation	WHITEsmile XTRA 38% HP, Germany	~8	In-office (with laser activation)	12 minutes every session, total 3 sessions
Group 4: control (no agent)	Not applicable	7.0	Not applicable	Not applicable

**Table 2:** Statistical differences of the test groups in the end of 4th, 8th, 12th, and 16th days.

Materials	Days 1–4	Days 6–8	Days 9–12	Days 13–16	Total
38% HP with light versus 10% CP		*	**	*	*
38% HP with light versus 38% HP with laser diode				*	*
38% HP with light versus control	*	*	**	*	*
38% HP with laser diode versus 10% CP					
38% HP with laser diode versus control				*	*
10% CP versus control					

*Statistically significant differences between the groups for Bonferroni test (*p* < 0.05).

**Statistically significant differences between the groups for Dunnett *C* test (*p* < 0.05).
